# Cross‑cultural adaptation and validation of the Persian version of the new Knee Society Knee Scoring System (KSS)

**DOI:** 10.1186/s13018-023-04347-7

**Published:** 2023-11-13

**Authors:** Alireza Mirahmadi, Pooya Hosseini-Monfared, Shayan Amiri, Fatemeh Taheri, Mehrdad Farokhi, Reza Minaei Noshahr, Seyed Morteza Kazemi

**Affiliations:** 1 Bone, Joint and Related Tissues Research Center, Shahid Beheshti University of Medical Sciences, Tehran, Iran; 2 Department of Orthopedic, School of Medicine, Shohadaye Haftom-e-Tir Hospital, Iran University of Medical Sciences, Tehran, Iran

**Keywords:** Knee, Osteoarthritis, Total knee arthroplasty, Surveys and questionnaires, Reliability, Validity, Persian, Iran

## Abstract

**Background:**

The new Knee Society Knee Scoring System (KSS) has been widely used to assess the symptoms, satisfaction, expectations, and physical activities of patients who undergo total knee arthroplasty (TKA). KSS has been translated and validated into many languages but not Persian. The aim of this study was to translate and evaluate the validity and reliability of the Persian version of the new KSS.

**Methods:**

The Persian version of the new KSS was translated and culturally adapted according to international guidelines, including translation, back-translation, pre-testing, and expert committee review. A total of 142 patients scheduled to undergo TKA were included in this study and were asked to complete the Persian-KSS, Oxford Knee Score (OKS), and the Visual Analogue Scale (VAS) index both two weeks before the surgery and 6 months after the surgery. Face, content, and construct validity were evaluated to assess the validity of Persian-KSS.

**Results:**

The Persian-KSS was comprehensive, indicating that the Persian version of KSS was clear and easy to understand for Persian-speaking patients undergoing TKA. The reliability of the Persian-KSS, assessed by Cronbach’s alpha, was 0.894 and 0.800 for the pre- and post-operative stages, respectively. The intraclass correlation coefficient (ICC) assessed the test–retest reliability, which was 0.766 and 0.796 for the pre- and post-operative stages, respectively. The construct validity analysis of Persian-KSS demonstrated a statistically significant correlation between Persian-KSS and the OKS (*r* = − 0.935, *p*-value = 0.000 for the pre-operative stage, and *r* = − 0.809, *p*-value = 0.000 for the post-operative stage) and VAS index (*r* = − 0.401, *p*-value = 0.001 for the pre-operative stage and *r* = − 0.259, *p*-value = 0.029 for the post-operative stage).

**Conclusion:**

The Persian-KSS, developed after the translation and cross-cultural adaptation process, was proven to be a reliable and valid assessment measure for those who undergo TKA.

## Introduction

Total knee arthroplasty (TKA) is an effective surgical treatment for people with end-stage osteoarthritis (OA). In recent years, the average increase of TKAs in the USA has been 156%, which is expected to continue in the coming years [1]. Studies have also found a growing demand for TKA in Iran [2]. Evaluating a patient's pain and function before and after TKA is necessary; therefore, various scoring criteria such as Oxford Knee Score (OKS), Knee injury and osteoarthritis outcome score (KOOS), Western Ontario and McMaster Universities Osteoarthritis Index (WOMAC), among many others, have been developed to evaluate the patient's satisfaction and function. The Knee Society Clinical Rating System was developed in 1989, consisting of clinical and subjective knee scoring [3]. In 2011, the new Knee Society Knee Scoring System (KSS) was developed to improve its validity and reliability, considering the patients' evolving expectations and functional needs [4]. The KSS consists of two parts, and doctors and patients provide input on this questionnaire, which thoroughly evaluates a patient's state and improvement after TKA.

The new KSS is a measure that evaluates the satisfaction, expectations, physical activities, and clinical and functional status of patients both in pre-operative and post-operative stages. It has four subscales: symptoms (consisted of three questions and scored out of 25), satisfaction (consisted of five questions and scored out of 40), expectation (consisted of three questions and scored out of 15), and activity (consisted of 19 questions and scored out of 110). The functional activity score is further divided into walking and standing, standard activities, advanced activities, and discretionary activities. A higher score in KSS indicates a better health state and better outcome [4].

When measuring health status, it is crucial to take culture into account because the way in which they experience and communicate symptoms of the health condition may vary based on their cultural heritage. The original English version of the KSS has been translated and validated in multiple languages [5–12]. However, it has not been translated and validated in Persian. In this study, we aimed to evaluate the validity and reliability of the Persian translation of the new Knee Society Knee Scoring System (Persian-KSS).

## Materials and methods

### Study design

This study was carried out in the Akhtar Orthopedic Hospital, including 142 patients who were scheduled for TKA. The study's inclusion criteria were Persian literate patients aged 18 years and above who were considered for primary TKA due to knee arthritis. The exclusion criteria of the study were: having TKA surgery due to trauma and following periarticular and articular fractures, patients with septic arthritis or rheumatoid arthritis, patients with poor cognitive function or poor reading skills, patients with neurological disorders that can affect mobility, patients with a history of knee surgery, and patients who were planned for revision surgery. Informed consent was obtained from all the participants, and the ethics committee of Shahid Beheshti University of Medical Sciences approved the study protocol (IR.SBMU.RETECH.REC.1402.240).

It has been demonstrated that the sample size for questionnaire validity studies depends on different factors, including the type of condition that is being assessed and the type of people who are participating in the study [13, 14]. It is recommended that the sample size should be decided based on similar previous studies [14]. We determined our sample size based on the average sample size of the validation studies of KSS in other languages, which are summarized in Table 8 and the formula described by Walter et al. [15]. The sample size was determined to be 133 with a significance level (*α*) of 0.05, power of 0.90, and expected reliability of 0.85 based on previous studies. Considering a 5% dropout rate, a total of 140 patients were needed for this study.

The patient's socio-demographical characteristics were recorded. Patients were asked to answer the pre-operative version two weeks before the surgery and the post-operative version 6 months later. All participants completed the Persian versions of the OKS, the Visual Analogue Scale (VAS) index, and the new KSS. The new KSS is a measurement that assesses patient satisfaction, expectations, physical activity, clinical status, and functional status both before and after surgery. We opted for the Persian OKS since it is an accurate index that clinically measures the pain and physical activity related to the knee, and it has been validated for knee OA patients [16].

### Translation and cross-cultural adaptation

The translation and cross-cultural adaptation of the questionnaire were done according to the recommendations of the international guidelines and considering the different lifestyles and cultures [17, 18].

The original English version of the questionnaire was translated into Persian independently by two medical doctors who were native Iranians and were fluent in both Persian and English. Next, another independent reviewer evaluated the translated versions to resolve the possible discrepancies. Finally, no significant difference between the two translated versions was observed. The Persian draft was back-translated into English by two native English translators who were blind to the original questionnaire. Afterward, two back-translation versions were compared to each other and to the original questionnaire by an independent reviewer, and no discrepancies were found.

After this process, a pilot study was conducted to pre-test these prototype versions of KSS for pre- and post-operative stages on 40 patients, and they were asked about its comprehensibility and appropriateness. Then, the final version of Persian-KSS was created after some adjustments.

### Reliability

Reliability is the instrument's capability to consistently reproduce a result in time and space [19]. Internal consistency, which is the homogeneity of the questions within a questionnaire, was assessed using Cronbach's alpha coefficient and calculated separately for the four subscales of the questionnaire (symptoms, patient satisfaction, patient expectations, and functional activities). Alpha values between 0.70 and 0.95 are considered an adequate consistency [20].

Regarding test–retest reliability, for both pre- and post-operative stages, 40 patients were asked to complete the questionnaire again after two weeks after completing the questionnaire for the first time. Test–retest was done to prove the results' consistency and stability of reliability. The patients received no additional interim treatment in these two weeks. The intraclass correlation coefficient (ICC) was utilized to evaluate the test–retest reliability of the Persian-KSS. Satisfactory and excellent reliability were defined as correlation coefficient values over 0.40 and 0.80, respectively [21].

### Validity

Validity refers to the ability to measure exactly what it proposes [20]. Several validity methods have been described, including face, content, and construct validity [22].

#### Face validity

Face validity is a subjective assessment of the operationalization of a construct. The questionnaire design is evaluated to see if it is consistent with the study's goals [23]. Ten orthopedics professionals were consulted for their opinions on the face validity of the Persian-KSS. The face validity is reported as the percentage of agreements on the face validity features of the Persian-KSS. The percentage between 80 and 90 indicates moderate agreement, and > 90 percent indicates full agreement about the face validity of the questionnaire [24].

#### Content validity

The assessment of content validity involved examining the ceiling and floor effects. This evaluation aimed to gauge the instrument’s sensitivity to detect various clinical outcomes and determine the percentage of patients achieving the highest and lowest scores. Scholars have indicated that an effect size of less than 15% is considered satisfactory evidence of the questionnaire's content validity [21].

#### Construct validity

Construct validity of the scale was assessed by calculating Spearman's correlation coefficient between the subscales of the Persian-KSS and the OKS and VAS index. The following guidelines were used to interpret the correlation coefficients (*r*): mild correlation (*r* < 0.3), moderate correlation (0.3 < *r* < 0.6), and strong correlation (*r* > 0.6) [25]. Convergent and divergent, also known as discriminant, validity was used to assess the construct validity in this study.

- Confirmatory factor analysis

Confirmatory factor analysis is a validity assessment method for the determination of whether the questions in a questionnaire have been selected properly. It is performed by structural equation modeling. The model fit was considered acceptable if two of the following three conditions were met: *p*-value > 0.05, root mean square error of approximation (RMSEA) values < 0.08, and relative Chi-square < 3 [26, 27].

- Convergent validity

Convergent validity is a strong correlation between the questions and the relevant domain. Convergent validity was assessed by the average variance extracted (AVE) and composite reliability (CR). Convergent validity exists if AVE > 0.5 and CR > 0.7 [28].

- Discriminant validity

Discriminant validity can be inferred when there is a lack of convergence between the scores of measures of different constructs. As a result, it tells us if scores from a construct's measure are distinct from other constructs or not. The discriminant validity of the Persian-KSS was determined by the heterotrait–monotrait (HTMT) ratio of correlations. HTMT is the average of the heterotrait–heteromethod correlations relative to the average of the monotrait–heteromethod correlations [29].

### Statistical analysis

Quantitative variables were presented as mean ± standard deviation (SD), and qualitative variables were presented as percent (%). The normality of the data distribution was assessed using the Kolmogorov–Smirnov test. Spearman’s correlation coefficient was used to assess the correlation of subscales of the Persian-KSS and the OKS and VAS index. Convergent validity was assessed by the average variance extracted values of the subscales of the Persian-KSS. The HTMT ratio determined discriminant validity. Statistical analyses were done using IBM SPSS statistics v26.0 (SPSS Inc, Chicago, IL) and AMOS v26.0 (SPSS Inc, Chicago, IL) for Windows. *p* value < 0.05 was considered statistically significant.

## Results

A total of 142 patients who met the inclusion criteria were studied. All the participants completed the questionnaire both in the pre- and post-operative stage. No complaints or difficulties were found regarding the comprehension of the Persian-KSS. The mean age of the patients was 66.39 ± 8.58. The demographic data of the patients are summarized in Table 1. The mean value of the Persian-KSS for each subscale is summarized in Table 2.

**Table 1:** Demographic data of the sample group (*n* = 142)

Characteristics	Value
Age, year, mean ± SD	66.39 ± 8.58
*Gender, n *(*%*)	
Male	34 (23.9%)
Female	108 (76.1%)
Height	162.29 ± 8.89
Weight	76.49 ± 17.10
BMI	28.94 ± 5.84

BMI, body mass index

**Table 2:** The values of the Persian-KSS

Domains of the Persian-KSS	Pre-operative (mean ± SD)	Post-operative (mean ± SD)	*p*-value
Symptoms (scored out of 25)	4.87 ± 3.121	18.65 ± 4.564	**0.000**
Satisfaction (scored out of 40)	9.83 ± 11.503	25.36 ± 7.075	**0.000**
Expectations (scored out of 15)	10.13 ± 2.223	10.00 ± 1.534	0.696
Activity (scored out of 110)	29.35 ± 21.706	63.58 ± 15.705	**0.000**
Total (scored out of 190)	49.03 ± 28.823	104.00 ± 21.834	**0.000**

KSS, Knee Society Score

Bold values indicate significance level which is considered* p*-value < 0.01

### Translation

The Persian-KSS was comprehensive in the pilot study; indicating that the Persian version of KSS was clear and easy to understand for Persian-speaking patients undergoing TKA. No major differences were found between the forward and backward translations and the original KSS for both pre- and post-op versions.

### Reliability

The Cronbach's alpha coefficient for the pre-operative Persian-KSS and post-operative Persian-KSS was 0.894 and 0.800, respectively, which indicates good consistency.

Test–retest evaluation of the new KSS was performed by calculating the ICC coefficient. The ICC score for pre-operative Persian-KSS was 0.766 (95% CI 0.664–0.848) and for post-operative Persian-KSS was 0.796 (95% CI 0.708–0.868), which indicates good test–retest reliability.

### Validity

#### Face validity

Face validity of the Persian-KSS was assessed by the percentage of agreement about the face validity features of the questionnaire by ten orthopedics specialists. The Persian-KSS had 96% agreement of the face validity, indicating full agreement about the Persian-KSS’s face validity.

#### Content validity

Content validity was evaluated through the floor and ceiling effects. The maximum score was obtained from two patients for the symptoms subscale (1.4%), three for the satisfaction subscale (2.1%), two patients for the expectations subscale (1.4%), and no patients for the activity subscale. The minimum scores were examined for floor effect. The minimum score was obtained from 10 patients for the symptoms subscale (7.0%), 32 for the satisfaction subscale (22.53%), and no patients for the expectations and activity subscale. There was no ceiling or floor effect for the total value of the Persian new KSS.

#### Construct validity

Regarding construct validity, Persian-KSS and its subscales correlate statistically with OKS (*r* = − 0.935, *p*-value = 0.000 for the pre-operative stage and *r* = − 0.809, *p*-value = 0.000 for the post-operative stage). Correlation coefficients between the subscales of the Persian-KSS and OKS are presented in Table 3.

**Table 3:** Spearman correlation between OKS and subscales of the Persian-KSS

	Spearman’s correlation coefficients	*p*-value
Symptoms	Pre-operative = − 0.440	**0.000**
	Post-operative = − 0.334	**0.004**
Satisfaction	Pre-operative = − 0.723	**0.000**
	Post-operative = − 0.560	**0.000**
Expectations	Pre-operative = 0.084	0.489
	Post-operative = 0.182	0.135
Activity	Pre-operative = − 0.845	**0.000**
	Post-operative = − 0.774	**0.000**
Total	Pre-operative = − 0.935	**0.000**
	Post-operative = − 0.809	**0.000**

Bold values indicate significance level which is considered* p*-value < 0.01

Also, the Spearman correlation test was run to determine the association between Persian-KSS and VAS score, which revealed a statistically significant correlation between VAS score and both pre-op and post-op Persian-KSS (*r* = − 0.401, *p*-value = 0.001 for the pre-operative stage and *r* = − 0.259, *p*-value = 0.029 for the post-operative stage).

- Confirmatory factor analysis

Confirmatory factor analysis (CFA) was also carried out as a measure of the construct validity of the Persian-KSS. The model-fit measures were used to assess the model’s goodness of fit, and the values were acceptable (Table 4).

**Table 4:** fit indexes of confirmatory factor analysis

Items	Pre-operative	Post-operative	Acceptable level
*p*-value	0.53	0.072	> 0.05
RMSEA	0.65	0.061	< 0.08
CMIN/DF	1.296	1.259	< 3
CFI	0.975	0.966	> 0.9
GFI	0.869	0.851	> 0.9
TLI	0.965	0.955	> 0.9

RMSEA, root mean square error of approximation, ratio of Chi-square minimum and degree of freedom; CFI, comparative fit index; GFI, goodness-of-fit index; TLI, Tucker–Lewis index

(b) Convergent validity

The convergent validity of the scales of the Persian-KSS was estimated using AVE. All Persian-KSS constructs, both pre- and post-operatively, had average variance extracted values greater than the threshold value of 0.50 [30]. Table 5 summarizes the AVE and composite reliabilities of the constructs of the Persian-KSS.

**Table 5:** Composite reliability and average variance extracted

Items	Pre-operative		Post-operative	
	CR	AVE	CR	AVE
Symptoms	0.742	0.574	0.857	0.776
Satisfaction	0.972	0.872	0.912	0.684
Expectations	0.663	0.451	0.894	0.930
Activity	0.737	0.441	0.504	0.264

CR, composite reliability; AVE, average variance extracted

(c) Discriminant validity

The heterotrait–monotrait (HTMT) ratio was used to evaluate the discriminant validity of the Persian-KSS since it has been demonstrated to be more sensitive than the formerly used Fornell–Larcker criterion [29]. In our study, all HTMT ratios—both pre-operative and post-operative—were below the necessary level of 0.85 [29]. Therefore, the discriminant validity was established. The results of discriminant validity assessment in the pre-operative and post-operative study are presented in Tables 6 and 7, respectively.

**Table 6:** Discriminant validity of the pre-operative Persian-KSS

Subscales of Persian-KSS	Activity	Expectations	Satisfaction	Symptoms
Activity				
Expectations	− 0.301			
Satisfaction	0.70301	− 0.4326		
Symptoms	0.39089	0.13269	0.33168

**Table 7:** Discriminant validity of the post-operative Persian-KSS

Subscales of Persian-KSS	Activity	Expectations	Satisfaction	Symptoms
Activity				
Expectations	0.131			
Satisfaction	0.065	0.012		
Symptoms	0.120	0.032	0.012	

## Discussion

In the present study, the Persian version of the new KSS was proved to be valid and reliable for assessing Persian-speaking patients undergoing TKA. No difficulties with comprehension were found in the translation and cross-cultural adaptation of the Persian-KSS. The percentage of female patients in this study (76.1%) was consistent with the known demographic characteristics of the Iranian population undergoing TKA [2].

In the Turkish version of KSS, “ballet” in the discretionary activity was replaced by “folk dance” [7]. In the Japanese version, “hiking” and “ground golf” were added to the questionnaire during the cross-cultural adaptation process [10]. In our study, no changes were applied to the activity section of the questionnaire since the activities were usual in Iran.

In our study, floor or ceiling effects were less than 10% in all the subscales, except the satisfaction subscale, which was 22.53%. In the Spanish version, ceiling effects were observed for the expectation subscale (38.64%) and floor effects for the advanced activity subscale (30.68%) [5]. A ceiling effect was observed in the expectations subscale in the French version of the KSS [12]. No floor or ceiling effects were reported in the Chinese, Japanese, Turkish, or German versions of KSS [6, 7, 10, 11]. Floor or ceiling effects were not evaluated in the Korean and Brazilian versions of KSS [8, 9].

The Persian-KSS was found to be reliable both in the pre- and post-operative stages for the patients who have undergone TKA. The Cronbach's alpha was consistent with KSS in other languages, which was reported from 0.71 to 0.94 (Table 8) [5–12]. The ICC score was used to assess the test–retest reliability of the Persian-KSS, which was acceptable and comparable with other versions of the KSS. The ICC score was reported to range from 0.65 to 0.95 for the KSS in other languages (Table 8) [5–12]. The time interval for test–retest evaluation could be the reason for the different ICC scores in different studies. The test–retest was performed at two weeks in our study, whereas in the studies of the Japanese and Chinese versions, this time was one week, and in the study of the Korean version, patients were asked to complete the questionnaire four weeks later [8, 10, 11].

**Table 8:** Summary of KSS validation studies in different languages

Author	Year	Sample size	Language	Cronbach’s alpha	ICC	Validity analysis
Debette et al	2014	80	French	0.7–0.9	0.87–0.97	KOOS, AMIQUAL, and SF-12
Liu et al	2015	105	Chinese	0.71–0.85	0.709–847	OKS, VAS, and SF-36
Hamamoto et al	2015	93	Japanese	0.78–0.94	0.65–0.88	OKS and SF-36
Silva et al	2017	90	Brazilian Portuguese	0.312–0.854	0.807–0.969	–
Kim et al	2017	123	Korean	0.83–0.92	0.69–0.86	WOMAC and SF-36
Özden et al	2019	66	Turkish	0.814–0.997	0.79–0.95	WOMAC and SF-36
Kayaalp et al	2019	133	German	0.78–0.94	0.82–0.97	WOMAC and SF-36
Ares et al	2021	176	Spanish	0.841–0.861	0.841–0.861	WOMAC and SF-12

The validity of Persian-KSS was determined by assessing the correlation with OKS and VAS index, which are valid and reliable scoring for the patients who undergo TKA [16]. A strong correlation was observed between the symptoms, activity, satisfaction, and total score of the Persian-KSS with the OKS. A similar correlation was observed in the studies of the Japanese and Chinese versions of KSS [10, 11].

Our study had a limitation since we used two different questionnaires, Persian-KSS and OKS, in the same session. This could cause a responder burden and potentially influence the patients' response. Also, we did not include revision TKA or patients with joint underlying diseases other than osteoarthritis. So, using this questionnaire to evaluate other patients is recommended.

## Conclusion

This study demonstrated that the Persian version of the new KSS is a valid and reliable questionnaire and can be used in all native Persian-speaking TKA patients both pre- and post-operatively.

## Data Availability

Please contact the authors for data requests.
